# The Global Healthcare Simulation Economy: A Scoping Review

**DOI:** 10.7759/cureus.22629

**Published:** 2022-02-26

**Authors:** Taylor Sawyer, Megan M Gray, Rachel Umoren

**Affiliations:** 1 Pediatrics, Seattle Children's Hospital, Seattle, USA; 2 Pediatrics, University of Washington School of Medicine, Seattle, USA; 3 Neonatology, Seattle Children's Hospital, Seattle, USA

**Keywords:** scoping review, economy, simulation, healthcare, global

## Abstract

Healthcare simulation is a technique that creates a situation or environment that allows persons to experience a representation of a real health care event for the purpose of practice, learning, evaluation, or gaining an understanding of systems or human actions. The use of healthcare simulation has grown rapidly over the last decade. In this review, we describe the global healthcare simulation economy. We reviewed the literature describing the global healthcare simulation economy using four research databases (Google Scholar, MEDLINE, Embase, and EconLit) as well as alternative sources. The specific aims were to examine the major economic themes facing the healthcare simulation economy. We found that the global healthcare simulation market is segmented based on product & services, fidelity, end-user, and geography. The market has experienced new player entry over the last few years, with most businesses focused in North America, Europe, and Asia-Pacific. The global healthcare simulation market is expected to reach between $3.19 and $7.7 billion by 2027, with a compound annual growth rate of 14.6% to 17.8%. Political and trade issues between America and China may increase the cost of goods in the short term. There are no global regulations on the use of healthcare simulation for training, licensing, or certification. Therefore, individual countries, states, and healthcare specialties establish individual regulations. We conclude that the major economic issues facing the global healthcare simulation economy include market segmentation, the entry of new players, and differential global growth. These factors, plus recent political and trade issues, and lack of regulations, could impact decision-making.

## Introduction and background

Healthcare simulation is a technique that creates a situation or environment that allows persons to experience a representation of a real health care event for the purpose of practice, learning, evaluation, or gaining an understanding of systems or human actions [1]. Simply put, healthcare simulation is the experiential learning every healthcare professional needs but cannot engage in during real-life patient care. The use of simulation is associated with improvements in healthcare provider knowledge and skills [2]. Participation in healthcare simulation is also associated with improvements in clinical care and patient outcomes [3,4].

Based on its proven benefits, the use of healthcare simulation products and services has grown rapidly over the last decade. This has caused a global expansion in both the demand and supply of healthcare simulation products and services. Understanding how the global healthcare simulation market's expansion has impacted healthcare simulation economics is essential for decision-makers. However, healthcare simulation's global economics and its impact on business decision-making have not been examined in detail.

In this research paper, we sought to describe the global healthcare simulation economy. To examine the topic, we performed a scoping review of the literature. Our specific aims were to examine the major economic issues facing the healthcare simulation economy and to investigate the influence of political, legal, regulatory, and technological issues. Based on this data, we explore how those issues impact decision-making by key stakeholders.

## Review

Search strategy

We created a search protocol using the Preferred Reporting Items for Systematic Reviews and Meta-analysis extension for Scoping Reviews (PRISMA-ScR) [5]. We searched the medical and business literature using search terms germane to the global healthcare simulation economy. The search strategy included the following keywords and their Boolean operators: healthcare, simulation, economy, and global. The search string used was (‘healthcare’/exp OR healthcare) AND (‘simulation’/exp OR simulation) AND (‘economy’/exp OR economy) AND (‘global’/exp OR global). The search was restricted to English language publications from 1 January 2000 to 28 February 2021. The search included four research databases: Google Scholar, MEDLINE, Embase, and EconLit. The search included examining source article citation and ‘gray literature,’ such as internet pages, market economic projections, corporate websites, meeting abstracts, and blogs.

Synthesis of results

Two authors screened all source content titles and abstracts for inclusion. The article’s abstract was evaluated for quality and fit with the research aims for titles that seemed worthy of inclusion. If the article was applicable, qualitative and quantitative data was extracted, and then the content was analyzed and categorized [6]. Information and content from the selected publications were then categorized and grouped into major themes. When available, relevant numeric data was tabulated or presented in graphic format for easier interpretation.

Results

**Figure 1:**
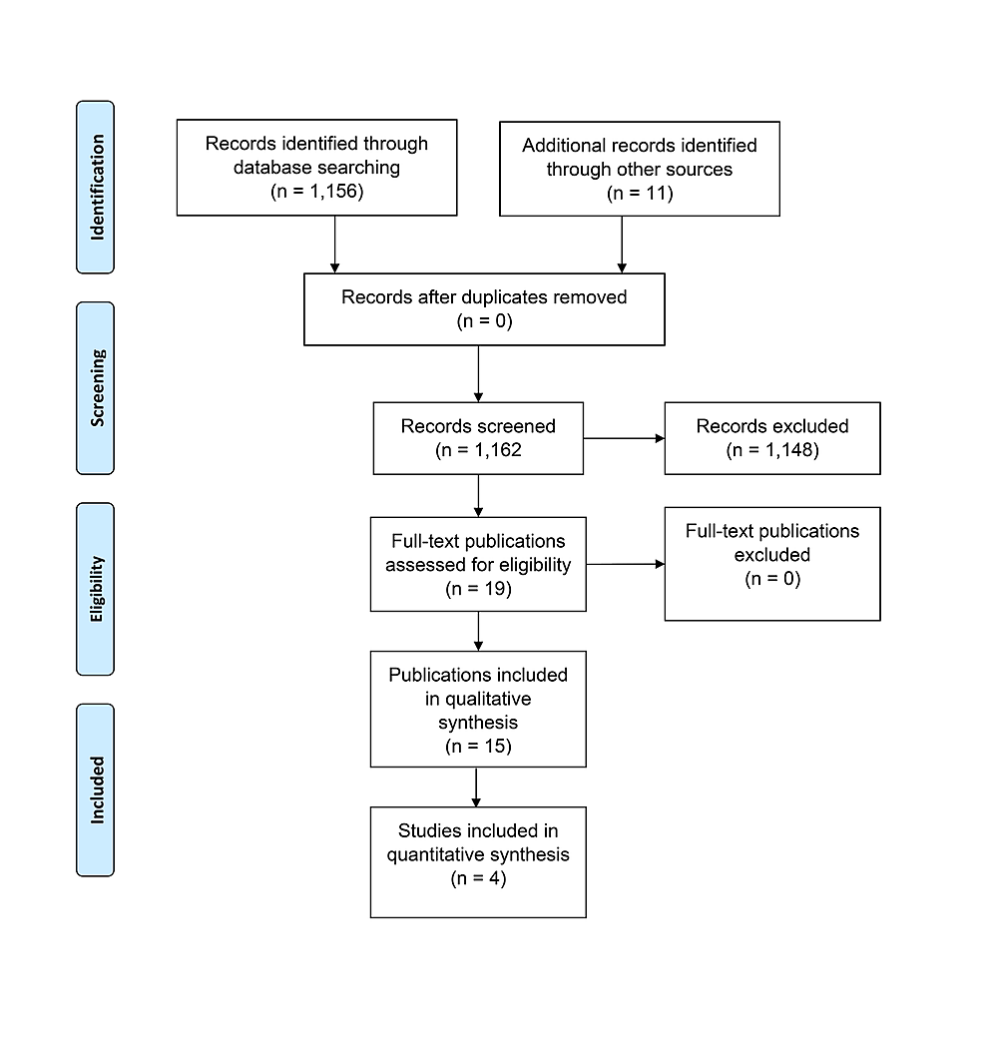
Study flow diagram

We identified 1,161 articles through database searching and from other sources. Of those, 19 directly related to our research aims and were included in either the qualitative or quantitative analysis (Figure 1). After a thorough review of the identified reports, we identified six major themes. The themes included 1) healthcare simulation stakeholders, 2) industries involved in healthcare simulation, 3) healthcare simulation market sectors, 4) healthcare simulation economic growth, 5) international political and trade issues, and 6) regulatory issues.

Healthcare simulation stakeholders

As part of the Research Into Global Healthcare Tools (RIGHT) project, Brailsford et al. developed a comprehensive list of potential healthcare simulation stakeholders [7]. Although the stakeholders’ list focused on the U.K. National Health Service, the roles could be extrapolated outside the U.K. system and applied globally. A list of healthcare simulation stakeholders adapted from those identified by Brailsford et al. [7] is provided in Table 1.

**Table 1:** Healthcare Simulation Stakeholders Adapted from [7].

Category	Example
Government	Health ministries, Department of Education, Department of Trade & Industry, Treasury
Civil services	Social care, Medicare, Medicaid
Public providers	National Health Service, Veterans Administration
Private providers	Private practice hospital and providers
Professional groups	British Medical Association, American Medical Association, Royal College
Professionals	Physicians, nurses, respiratory therapists, etc.
Healthcare users	Patients, families, caregivers
The public	Taxpayers

Industries involved in healthcare simulation

The Global Industry Classification Standard (GICS) is a method to assign companies to sectors, industry groups, industries, and sub-industries that best define their business [8]. The use of the GICS creates a common language among global market participants. Currently, GICS includes 11 sectors, 24 industry groups, 69 industries, and 158 sub-industries. A list of selected GICS industries involved in healthcare simulation and specific examples of products and services within the industry are shown in Table 2.

**Table 2:** Industries in Healthcare Simulation * Global Industry Classification Standard industries [8].

Industry*	Example product and service
Electrical Equipment	Electrical components and equipment used in simulation manikins
Professional Services	Human resource and employment services used to hire Simulation Specialists; Healthcare simulation research and consulting services
Diversified Consumer Service	Healthcare simulation education services and professional societies and organizations
Internet & Direct Marketing Retail	Healthcare simulation company website and online retail space
Distributors	Healthcare simulation equipment distributors
Health Care Equipment & Supplies	Healthcare equipment used in simulation training (e.g., gurney, stethoscope, IV equipment, etc.)
Media	Advertising for simulation equipment and services; healthcare simulation journal and book publishing
Electronic Equipment, Instruments & Components	Electronic components and instruments used in simulation manikins
Technology Hardware, Storage & Peripherals	Audiovisual hardware used in a simulation center
Software	Healthcare simulation application software; software systems used to run Healthcare simulation applications

Healthcare simulation market segmentation

The global healthcare simulation market can be segmented in several ways. In a 2020 report, Verified Market Research segmented the global healthcare simulation market into products and services, end-users, geography, and key players [9]. A report from Business Insights in 2020 also included the segment of end-users [10]. Based on these sources, the global healthcare simulation market can be sub-segmented within these five broad categories, as seen in Table 3.

**Table 3:** Healthcare Simulation Market Segmentation *Select companies based on market share. Adapted from [9] and [10].

Segment	Sub-segments
Product and service	Healthcare anatomical models
	Web-based simulation
	Healthcare simulation software
	Simulation training services
Fidelity	Low-fidelity
	Medium-fidelity
	High-fidelity
End-user	Academic institutions
	Hospitals & clinics
	Military organizations
	Others
Geography	North America
	Europe
	Asia-Pacific
	Latin America
	Middle East
	Africa
Key Players*	Laerdal Medical
	Canadian Aviation Electronics, Ltd.
	Gaumard Scientific Company, Inc.
	Surgical Science Sweden AB
	3D Systems, Inc.
	Limbs & Things, Ltd.

Healthcare simulation economic growth

Simulation is a small part of the healthcare field. The GICS does not list healthcare simulation as an industry or sub-industry within the “Health Care” sector [8]. However, the increasing focus on patient safety in the healthcare field is expected to boost the healthcare simulation market’s growth over the next decade [9]. In recent years, the healthcare simulation market has seen the entry of many new players. In this market composed of many small and medium players, the competition is high [11].

**Figure 2:**
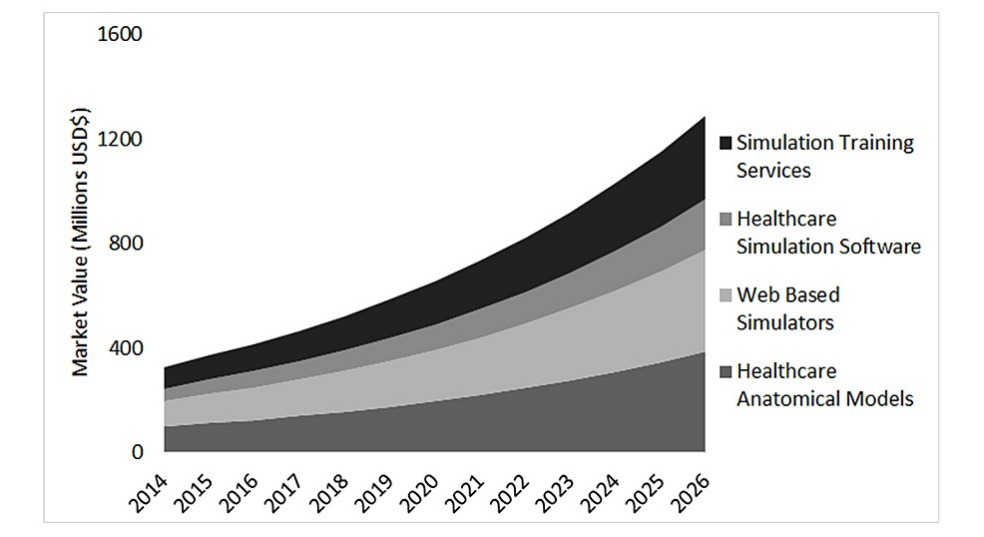
Projected healthcare simulation growth in the U.S.: 2014 to 2026 Adapted from [13].

In 2019, the global healthcare simulation market was estimated at $1.42 to 2.27 billion [9]. The market is projected to reach somewhere between $3.19 and $7.7 billion by 2027, with a compound annual growth rate (CAGR) of 14.6% to 17.8% from 2020 to 2027 [9, 12]. Figure 2 provides a graphic representation of healthcare simulation growth in the U.S. from 2014 projected to 2026 [13].

In a market analysis, Grand View Research found that in the healthcare simulation market segment of Product & Service, the model-based sub-segment generated the highest revenue in 2019 of $727.47 million [14]. However, the web-based simulation segment was expected to see the highest compound annual growth rate (CAGR) of 15.2% during the period from 2020 to 2027 [14]. The low-fidelity simulators segment had the highest market share in 2019, with $671.3 million in the fidelity segment. However, the high-fidelity segment is expected to see the highest CAGR of 15.3% from 2020 to 2027 [9]. As seen in Table 3, the global medical simulation market is spread across various regions. In 2019, North America accounted for nearly half of the market [10]. From 2020 to 2027, the Asia-Pacific market is expected to see the highest CAGR of 15.2% [10]. Regarding end-users, hospitals comprised more than half the market in 2019 with $761.4 million in spending; however, academic institutions are projected to have the highest CAGR from 2020 to 2027 at 15.3% [10].

Despite the rapid growth, there are several barriers to the use of simulation globally. In the developed world, barriers to healthcare simulation adoption include lack of funding, lack of time to participate in simulation exercises, and lack of management interest or buy-in [11]. Rishipathak et al. identified the lack of capital investment as a critical barrier to the use of healthcare simulation in the developing world [15]. Rishipathak et al. speculated that in resource-limited countries ‘Hub and Spoke’ model - wherein one simulation center in a defined geographical area caters to all healthcare institutes in its radius - could reduce costs [15]. Young et al. identified three challenges for simulation in healthcare. The barriers included: “how good is good enough,” how is modeling linked to the decision-making, and cultural barriers [16]. The cultural barriers to simulation vary from country to country.

International political and trade issues

As a global industry, healthcare simulation is impacted by international political and trade disputes. In recent years one trade issue that has impacted healthcare simulation is the U.S. and China trade war. The U.S. is the world’s largest medical technology importer. China is the world’s fourth-largest exporter [17]. In June 2019, the U.S. Trade Representative released a list of imports from China subject to a 25 percent tariff under Section 301 [18]. In total, the Section 301 tariffs impacted roughly $50 billion of Chinese imports to the U.S. each year and directly impacted nearly $1.8 billion of medical imports [19]. Those tariffs are anticipated to create a ripple effect that will increase costs in the U.S. healthcare systems. Varas and O'Neill Hayes estimated the tariffs would translate to price increases for medical equipment by roughly $400 million in the U.S. [19]. Fernandez estimated the tariffs plus the duties on imported steel and aluminum could result in a $600 to $800 million increase in costs for the healthcare industry [17]. According to Varas and O'Neill Hayes, both healthcare practitioners and consumers could shoulder the increased costs in acquiring medical equipment from China [19]. The direct impact of the Section 301 tariffs on healthcare simulation costs is unclear.

Regulatory issues

Simulation is widely used in certification programs, licensing, and the assessment for credentialing in medicine, nursing, and dentistry [20]. However, there are no global regulations on the use of healthcare simulation for certification, licensing, or credentialing. Therefore, simulation is regulated independently by each country and regions/states within the country. However, many countries' regulatory systems can pose a substantial barrier to the consistent use of simulation [20]. For example, in the U.S., there are 70 state medical boards and 24 specialty certification boards involved in physician certification.

In the U.S., some state Boards of Nursing (BONs) allow simulation to substitute for traditional clinical learning hours for nursing students. Bradley et al. found a significant amount of variability in the number of traditional clinical hours that can be replaced with simulation in prelicensure nursing education programs [21]. The authors felt that the variability in how state BONs define and regulate simulation in nurse training raises questions about learner outcomes' consistency [21]. A meta-narrative review by Roberts et al. found that while simulation is widely used as an adjunct to clinical hours, regulations need to be defined around the simulation modality used, the number of hours of simulation compared with clinical practice, and the assessment tools used to ensure quality [22].

For physicians, the American College of Surgery and the Association for Surgical Education have developed a simulation-based medical student surgical skills curriculum for medical school students [20]. Many American Board of Medical Specialties maintenance of certification (MOC) programs use simulation as part of the MOC process. Some examples include the American Board of Anesthesiology, the American Board of Internal Medicine, and the American Board of Family Medicine.

The U.K.’s National Health Service is developing a national strategy to ensure equity of access to healthcare simulation education and training across England [23]. The goal is to ensure healthcare simulation education in the U.K. provides value and delivers high-quality and patient-centered educational outcomes [23]. Creating national regulations and standards for the delivery of healthcare simulation education and training is part of that work [24].

Discussion

The results of this scoping review revealed several key themes about the global healthcare simulation economy. Healthcare simulation is a growth industry, and economic forces favor healthcare simulation producers and service providers in the short term. The web-based simulation and high-fidelity simulation market segments are projected to have the highest near-term growth within this growing economic sector. The Asia-Pacific market and academic institutions are predicted to have the highest short-term future demand. Recent trade disputes between the U.S. and China may increase healthcare simulation equipment costs in the U.S.; however, the overall impact is unclear. A lack of global regulation around healthcare simulation requires country and state-specific knowledge of the use of healthcare simulation for training, certification, and licensure.

There are several ways this data informs decision-making. Firstly, for producers of healthcare simulation goods and services, the data reported here provide evidence that future demand will be high. This anticipated high demand justifies the heavy near-term investment of resources in this sector. Market segmentation and differential global growth must be adjusted for, however, when making decisions on where to invest resources to reap the most gains. Secondly, for healthcare simulation goods and services consumers, the expected increase in supply should bring down overall costs. This anticipated drop in costs is potentiated by the rapid entry of new players into the healthcare simulation marketplace and the overall trend for decreasing technology costs over time. Therefore, future market cost considerations are important when considering the timing of significant monetary investments in healthcare simulation. Additionally, the recent international trade disputes between the U.S. and China could increase costs within the U.S. in the short term. Finally, knowledge of regulatory issues around healthcare simulation for training, licensure, and certification is vital for both producers and consumers. Local, state, or national regulations requiring healthcare simulation use would provide an incentive towards local market growth. On the contrary, international regulations could foster growth in areas not currently invested in healthcare simulation.

This report has some limitations. Like any literature review, the results presented are entirely dependent on the search performed. We tried to perform a systematic search of the literature using published methods and applicable search terms. However, many of the final reports used in this paper were identified through other sources and within the gray literature. It is possible that another researcher using different search terms and methods may arrive at different results. Also, we did not have a method to individually verify the financial data on current market size and projected future growth. Therefore, the accuracy of that data cannot be fully validated. We included professional simulation societies and trade organizations as part of the “Diversified Consumer Service” industry (Table 2). Such organizations - which include the Society for Simulation in Healthcare (SSH), the Society in Europe for Simulation Applied to Medicine (SESAM), the International Nursing Association of Clinical and Simulation Learning (INACSL), the International Pediatric Simulation Society (IPSS), and others - collect dues from members and provide services including educational courses, individual certification, and program accreditation. Given the important roles of these professional societies in the global healthcare economy, they may warrant further investigation. Finally, the major themes noted here are based on our interpretation and analysis of the reports identified. It is possible that other researchers could have identified different or additional themes.

## Conclusions

In conclusion, to summarize our findings, the major economic issues facing the global healthcare simulation economy include diverse stakeholders, market segmentation, the entry of new players, and rapid differential global growth. In addition to these factors, recent international political and trade disputes and changing regulations around healthcare simulation could impact business decision-making by key stakeholders.

This work is important to the field of healthcare simulation because the data presented here can help inform decision-making by both suppliers and consumers. Future research in the area of healthcare simulation economics is needed to more closely examine the market forces at play within individual countries. Also, further investigation into the impact of professional healthcare simulation societies may be helpful to fully understand their impact on a national and global scale.
